# Hepatitis B Infection Among Pregnant Women in China: A Systematic Review and Meta-Analysis

**DOI:** 10.3389/fpubh.2022.879289

**Published:** 2022-04-12

**Authors:** Dan Liu, Yan Liu, Jiaqi Ni, Hailong Li, Linan Zeng, Chuan Zhang, Li Zhang, Qin Yu, Bin Wu, Lingli Zhang

**Affiliations:** ^1^Department of Pharmacy/Evidence-Based Pharmacy Center, West China Second University Hospital, Sichuan University, Chengdu, China; ^2^Key Laboratory of Birth Defects and Related Diseases of Women and Children, Sichuan University, Ministry of Education, Chengdu, China; ^3^West China School of Pharmacy, Sichuan University, Chengdu, China; ^4^Departments of Obstetrics and Gynecology, West China Second University Hospital, Sichuan University, Chengdu, China; ^5^National Drug Clinical Trial Institute, West China Second University Hospital, Sichuan University, Chengdu, China; ^6^Medical Decision and Economic Group, Department of Pharmacy, Ren Ji Hospital, School of Medicine, Shanghai Jiao Tong University, Shanghai, China

**Keywords:** hepatitis B - infectious disease transmission, pregnant women, prevalence, China, systematic review, meta-analysis

## Abstract

**Background:**

A study of the current situation and characteristics of hepatitis B virus (HBV) infection among Chinese pregnant women is meaningful to provide baseline information for future research and policy making, with an aim to eliminate HBV in China.

**Objectives:**

To provide the epidemiological status of HBV infection among pregnant women in China.

**Methods:**

PubMed, EMbase, The Cochrane Library, and three Chinese databases were searched. Cohort studies and cross-sectional studies on HBV prevalence in Chinese pregnant women, published after 2016, were retrieved. In addition, combined HBV prevalence and 95% confidence interval (CI) were calculated. This research was registered in PROSPERO (CRD42021289123).

**Main Results:**

A total of 42 studies were included in the study, with a sample size of 4,007,518 cases, and 20 provinces in China. The prevalence of HBV in Chinese pregnant women was 6.64% (95% CI: 5.72–7.57%) during the period between 2016 and 2021. Among HBsAg positive pregnant women, the HBeAg positive rate was 25.80% (95% CI: 22.26–29.69%). Moreover, geographic regions with HBV prevalence ranking from high to low were in western China, eastern China, and central China, successively.

**Conclusion:**

The prevalence of HBV in Chinese pregnant women is intermediate endemic, although disparities exist between different regions. Among pregnant women with HBV infection, a high proportion of the patients have strong infectivity. Factors affecting HBV prevalence remain controversial, which demands further studies.

**Systematic Review Registration:**

https://www.crd.york.ac.uk/PROSPERO/, identifier: CRD42021289123.

## Background

Hepatitis B infection is caused by the hepatitis B virus (HBV), a leading cause of cirrhosis and hepatocellular carcinoma worldwide (1, 2). In 2016, World Health Organization (WHO) proposed the global health sector strategy on viral hepatitis control, which set the goal to eliminate viral hepatitis as a major public health threat by 2030 (3). One of the targets is to decrease the HBV prevalence in children under the age of 5 years to 0.1%. As mother-to-child transmission is the predominant mode of HBV transmission, blocking this pathway is an essential step to eliminate HBV (4, 5). One effective method is to standardize the health management of pregnant women with chronic HBV infection. China has the largest disease burden of HBV infection in the world (6). However, the current situation and characteristics of HBV infection among Chinese pregnant women remain unclear. Further studies are needed to support the formation of a health policy and future research to eliminate HBV. An observational study with a large sample size was recently conducted to evaluate the national and regional HBV prevalence among Chinese pregnant women (7). The results revealed that the HBV prevalence among pregnant women in China was intermediate endemic between 2015 and 2020, although disparities existed between different regions. Some limitations of that study include lack of demographic information of pregnant women included (such as age, occupation, times of gestation and parity), and information of other HBV serological markers (such as HBeAg, anti-HBs, anti-HBc, anti-HBe, and HBV DNA), which limited the possibility to explore the relationship between these factors and HBV prevalence. A systematic review and meta-analysis was conducted based on literature published after 2016 to provide a better understanding of HBV epidemiology in Chinese pregnant women.

## Methods

This systematic review and meta-analysis was conducted following the criteria of the PRISMA (Preferred Reporting Items for Systematic Reviews and Meta-Analyses) statement guidelines (8), and it was registered in PROSPERO (CRD42021289123).

### Search Strategy

Databases including PubMed, EMbase, The Cochrane Library, CNKI (Chinese National Knowledge Infrastructure), Wan-Fang Data, and VIP were searched for cohort studies and cross-sectional studies. In addition, the references of the retrieved studies were reviewed. Literature published between January 1, 2016 and June 1, 2021, reporting HBV prevalence in Chinese pregnant women, were included. A combination of subject words and free text search was used during the searching process. The example search terms are demonstrated in Table 1.

**Table 1:** The example of search terms (PubMed).

#1	(Pregnant Women[Title/Abstract]) OR (Pregnancy[Title/Abstract]) OR (“Pregnant Women”[Mesh]) OR (“Pregnancy”[Mesh])
#2	(Chinese[Title/Abstract]) OR (China[Title/Abstract]) OR (“China”[Mesh])
#3	(HBsAg[Title/Abstract]) OR (hepatitis B[Title/Abstract]) OR (Hepatitis B virus[Title/Abstract]) OR (HBV[Title/Abstract])) OR (Hepatitis B Surface Antigens[Title/Abstract]) OR (“Hepatitis B Surface Antigens”[Mesh])) OR (“Hepatitis B virus”[Mesh]) OR (“Hepatitis B”[Mesh])
#4	#1 AND #2 AND #3

### Inclusion and Exclusion Criteria

Inclusion criteria were as follows: (1) participants: pregnant women in China; (2) outcomes: HBV prevalence (HBsAg positive rate); (3) study designs: cohort studies and cross-sectional studies; (4) languages: English and Chinese. All articles were published between 2016 and 2021. The exclusion criteria included HBV prevalence in populations with specific diseases, such as women with intrahepatic cholestasis of pregnancy or preterm birth. If there was overlap in the sample source between different literature (such as participants recruited in the same hospital during the same time period), we included only the most updated report.

### Study Selection and Data Extraction

Two independent reviewers (LD and LY) screened articles, extracted data, and cross-checked results. When conflicts occurred, consensus was achieved after further discussion. Titles, abstracts, and full text were reviewed for relevance. Authors of the original studies were contacted by email or telephone for further information when necessary. The following data were extracted from literature: (1) general information of the studies (such as article title, main authors, publication time, study design, and the region where the study was conducted); (2) baseline characteristics of the study populations (such as sample size, age, education level, and occupation); (3) outcome indicators (such as the number of HBsAg positive individuals, HBsAg test methods, HBV serological markers, HBV DNA test results, and mother-to-child transmission rate); (4) key elements for risk of bias assessment.

### Risk of Bias Assessment

Two reviewers (LY and NJQ) independently assessed the risk of bias for the studies included and cross-checked the results. Cohort studies were assessed using the Newcastle Ottawa Scale (NOS) (9), and cross-sectional studies were assessed using the Agency for Healthcare Research and Quality (AHRQ) form (10).

### Data Analysis

Statistical analysis was conducted with R software. Data were synthesized using a random effect model in consideration of heterogeneity among studies. Moreover, individual proportions and the HBV pooled prevalence were assessed with 95% confidence interval (CI). Subgroup analysis and meta-regression were conducted based on the geographical region, economic level of the province, sample source (regions or hospitals), study designs, and study quality to detect the source of heterogeneity. Provinces in China were categorized into three groups based on the geographical regions: eastern China, central China, and western China. In addition, the economic level of the province was categorized as high level (ranks 1–10), medium level (ranks 11–20), or low level (ranks 21–31) based on the rank of per-capita gross domestic product (GDP) among provinces in China in 2020 (11). A sensitivity analysis was conducted by sequential removal of each study, in order to evaluate the individual study's impact on the overall pooled effect. Furthermore, studies with quality score ≤ 3 points were omitted to assess their impact on the overall pooled effect of studies with high risks of bias. Potential publication bias was assessed graphically by funnel plot and examined by Begg's and Egger's tests (significant when *p* < 0.05).

## Results

### Study Characteristics

**Figure 1:**
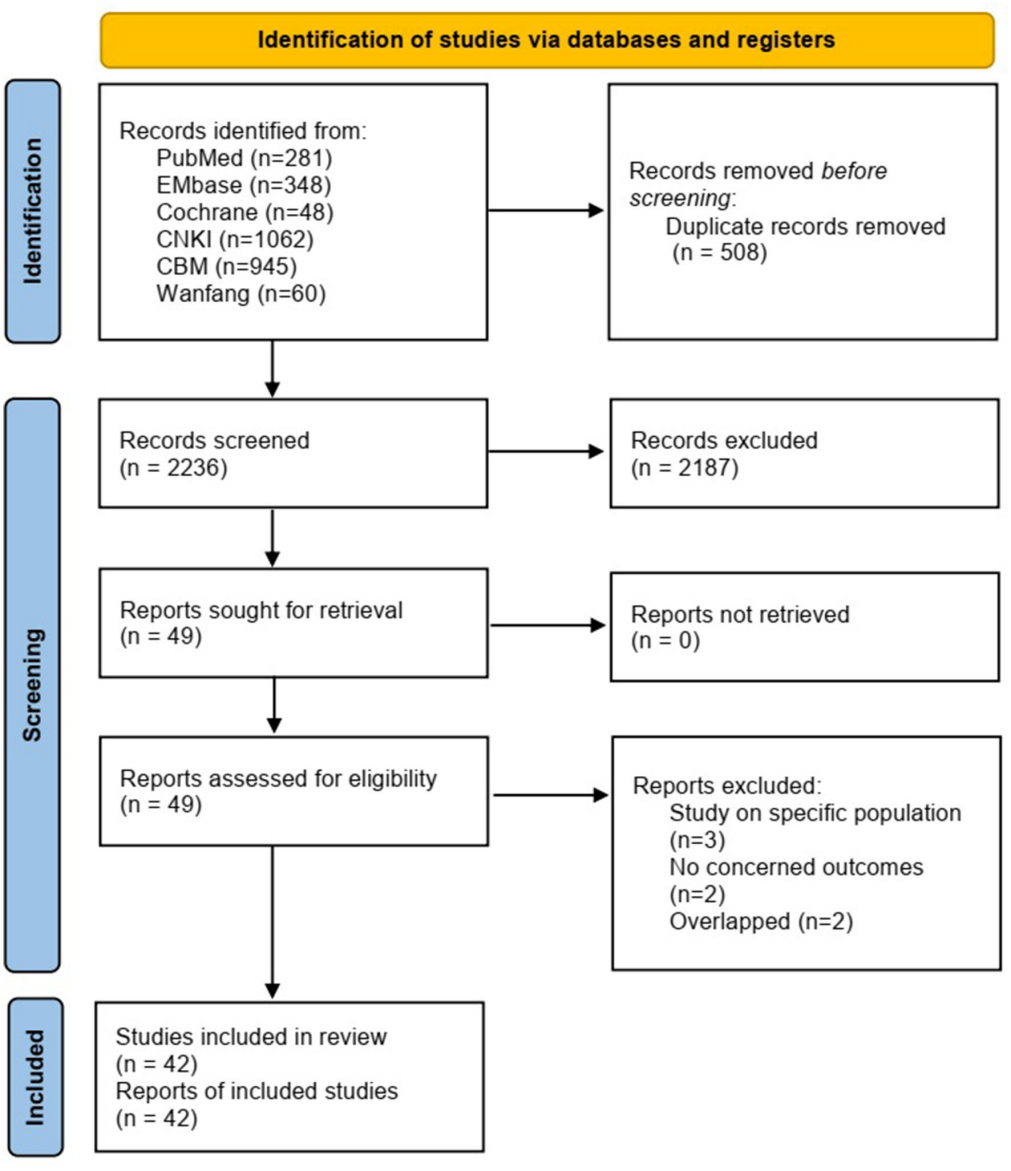
Flow diagram of study selection.

A total of 2,744 articles were retrieved initially. Following the screening of titles, abstracts, and full text, a total sample size of 4,007,518 pregnant women and 42 studies (12–53) were included in the meta-analysis (Figure 1). Seven cohort studies and 35 cross-sectional studies were included (Table 2). Generally, the studies included in the analysis were conducted in 20 provinces. The regional distributions included eastern China (19 studies), central China (6 studies), and western China (17 studies). The economic level was ranked as high level (24 studies), medium level (7 studies), and low level (11 studies). The sample size varied from 496 to 1,809,893 across studies.

**Table 2:** Characteristics of included studies.

**References**	**Design**	**Timespan**	**Province**	**Region**	**Sample source**	**Sample size**	**Positive HBsAg**	**Age (Mean±SD)**	**Labratory test**	**Quality score**
Sun et al. (32)	Cohort	2005–2017	Yunnan	Western	Medical	49,479	1,624	HBsAg positive: 29.6 ± 4.4 HBsAg negative: 29.7 ± 4.3	NR	7
Wen et al. (41)	Cross-sectional	2015–2019	Guangdong	Eastern	Regional	100,500	9,290	NR	ELISA	2
Du (17)	Cross-sectional	2016–2018	Hubei	Central	Medical	12,861	862	NR	ELISA	3
Liu et al. (28)	Cross-sectional	2013–2016	Yunnan	Western	Medical	15,641	1,234	Median: 28 (26–32)	ELISA	8
Wang et al. (39)	Cross-sectional	2014–2017	Jiangsu	Eastern	Medical	31,457	1,586	28.39 ± 5	CLIA, ECLIA, time-resolved methods	2
Zhang and Li (46)	Cohort	2009–2018	Fujian	Eastern	Medical	85,190	9,699	HBsAg positive: 30.33 ± 4.50	NR	7
Zhang et al. (48)	Cross-sectional	2015–2016	Henan	Central	Medical	1,870	92	27.12 ± 5.31	NR	7
Zhao et al. (49)	Cohort	2011–2018	Fujian	Eastern	Regional	33,437	3,789	HBsAg positive: 29.3 ± 4.3	NR	7
Zou et al. (53)	Cross-sectional	2016–2018	Jiangsu	Eastern	Regional	2,077	78	Range:19–42	Latex immunochromatography	3
Huang (23)	Cross-sectional	2014–2018	Guangxi	Western	Medical	2,861	277	27.5 ± 4.5	NR	3
Zhang et al. (47)	Cross-sectional	2017–2019	Tibet	Western	Regional	496	65	28.62	LAT	4
Cai et al. (13)	Cohort	2014–2015	Anhui	Central	Medical	3,329	346	HBsAg positive: 27 HBsAg negative: 24.3	ELISA, ECLIA	8
Guo et al. (19)	Cross-sectional	2016–2018	Hubei	Central	Medical	7,000	450	Range: 16–45	ELISA	3
He et al. (20)	Cross-sectional	2017	Jiangsu	Eastern	Regional	22,595	873	28.36 ± 4.46	ELISA	4
Kang (24)	Cross-sectional	2014–2018	Tianjin	Eastern	Medical	3466	133	28.76 ± 4.87	TRFIA	3
Tang et al. (35)	Cross-sectional	2014–2015	Guizhou	Western	Medical	2,118	119	26.4 ± 5.3	ELISA	6
Wang et al. (39)	Cross-sectional	2015–2017	Zhejiang	Eastern	Regional	43,628	2,168	NR	ELISA	3
Ying (43)	Cross-sectional	2015–2017	Zhejiang	Eastern	Regional	42,815	2,292	NR	LAT	4
Zhou (51)	Cross-sectional	2017–2018	Tibet	Western	Medical	2,136	192	28.96 ± 5.02	NR	7
Liu et al. (27)	Cross-sectional	2015–2017	Fujian	Western	Medical	14,760	1,221	NR	NR	3
Wang et al. (37)	Cross-sectional	2014–2016	Guangdong	Eastern	Medical	17,722	967	28.7 ± 3.4	ELISA	1
Wei et al. (40)	Cross-sectional	2012–2017	Sichuan	Western	Medical	131,507	9,722	NR	ELISA	5
Zeng et al. (44)	Cross-sectional	2017–2018	Jiangxi	Central	Medical	45,413	953	29.17 ± 5.04	ELISA	3
Ma et al. (29)	Cross-sectional	2016–2017	Qinghai	Western	Medical	60,027	1,912	NR	ELISA	2
Chen et al. (15)	Cross-sectional	2010–2015	Shaanxi Province	Western	Medical	13,451	951	Median:27(19–42)	CMIA	5
Sheng et al. (30)	Cross-sectional	2016	Liaoning	Eastern	Medical	14,314	441	31.1 ± 4.5	CMIA	6
Zhong (50)	Cross-sectional	2015–2017	Chongqing	Western	Medical	1,000	80	24.86 ± 2.55	ELISA	3
Wang et al. (36)	Cross-sectional	2015–2016	Guangdong	Eastern	Medical	13,093	1,424	29.38 ± 4.77	NR	5
Hu (21)	Cross-sectional	2014–2016	Chongqing	Western	Regional	359,570	19,237	NR	NR	6
Bao (12)	Cross-sectional	2011–2015	Guangdong	Eastern	Regional	732,783	63,495	27 ± 4	ELISA	6
Gong et al. (18)	Cross-sectional	2014–2016	Guizhou	Western	Medical	2,648	243	<35: 2,202 ≧35: 446	TRFIA	4
Zhuang et al. (52)	Cohort	2012–2016	Jiangsu	Eastern	Medical	37,264	1,115	Median: 26 (25–29)	ELISA	8
Liang et al. (26)	Cross-sectional	2014-2015	Guangdong	Eastern	Medical	6930	661	NR	CLIA	3
Sun et al. (31)	Cross-sectional	2013–2016	Shanghai	Eastern	Medical	63,109	2,924	20–50	CLIA	4
Huang et al. (22)	Cross-sectional	2013–2014	Fujian	Western	Medical	6,225	720	NR	NR	3
Tang et al. (34)	Cross-sectional	2014	Guangdong	Eastern	Regional	180,9893	182,782	NR	ELISA	3
Cui et al. (16)	Cohort	2012–2015	Jiangsu	Eastern	Medical	21,331	519	HBsAg positive: 27.59 ± 4.02 HBsAg negative: 27.03 ± 4.19	NR	7
Tan et al. (33)	Cohort	2009–2010	Sichuan	Western	Medical	22,374	948	Median: 27 (23–31)	ELISA	7
Wu (42)	Cross-sectional	2013–2015	Shanxi	Central	Medical	16,770	160	NR	ELISA	4
Li et al. (25)	Cross-sectional	2008–2014	Guangdong	Eastern	Medical	22,906	2,164	NR	NR	5
Zhang et al. (45)	Cross-sectional	2014–2015	Guizhou	Western	Medical	18,007	517	25.7	ELISA	3
Chen et al. (14)	Cross-sectional	2015	Guangxi	Western	Regional	115,484	8,751	NR	ELISA	4

*ELISA, Enzyme-linked immunosorbent assay; NR, not report; HBsAg, hepatitis B surface antigen; ECLIA, electrochemiluminescence immunoassay; TRFIA, time resolved fluoroimmunoassay; CMIA, chemiluminescence micro particle immunoassay; CLIA, chemiluminescence immunoassay; LAT, latex agglutination test*.

In terms of quality, all cohort studies included had a total quality score ≥ 7 points, with 5 studies achieving 7 points and 2 studies achieving 8 points (Supplementary Table S1). For cross-sectional studies, 17 studies had a quality score ≤ 3 points, 15 studies had 4–6 points, and 3 studies had ≥ 7 points (Supplementary Table S2). Most cross-sectional studies did not “explain any patient exclusions from analysis,” “describe how confounding was assessed and/or controlled,” or “explain how missing data were handled in the analysis.”

### Prevalence of HBV Infection in Pregnant Women

**Figure 2:**
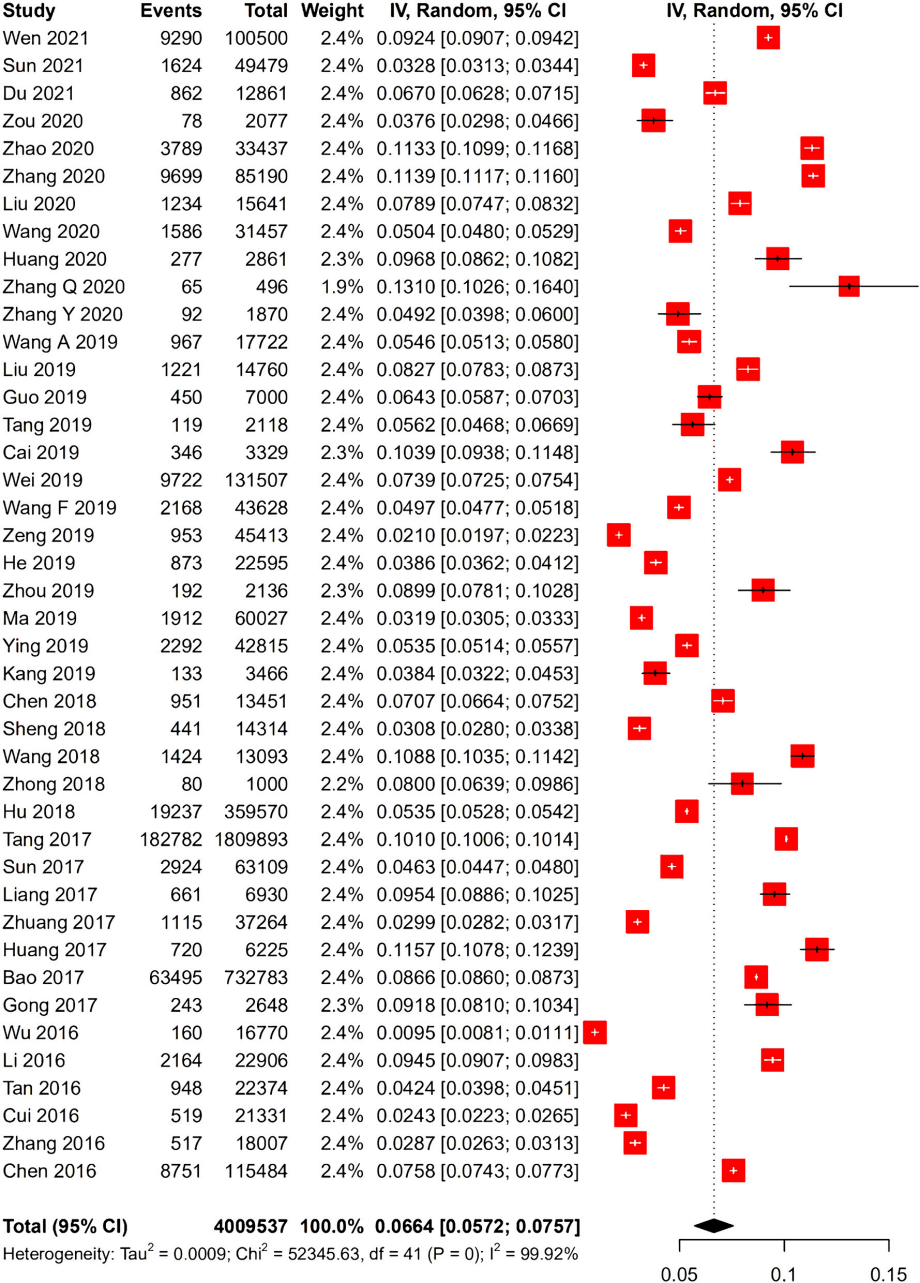
Forest plot of hepatitis B virus (HBV) infection prevalence rate in Chinese pregnant women.

**Figure 3:**
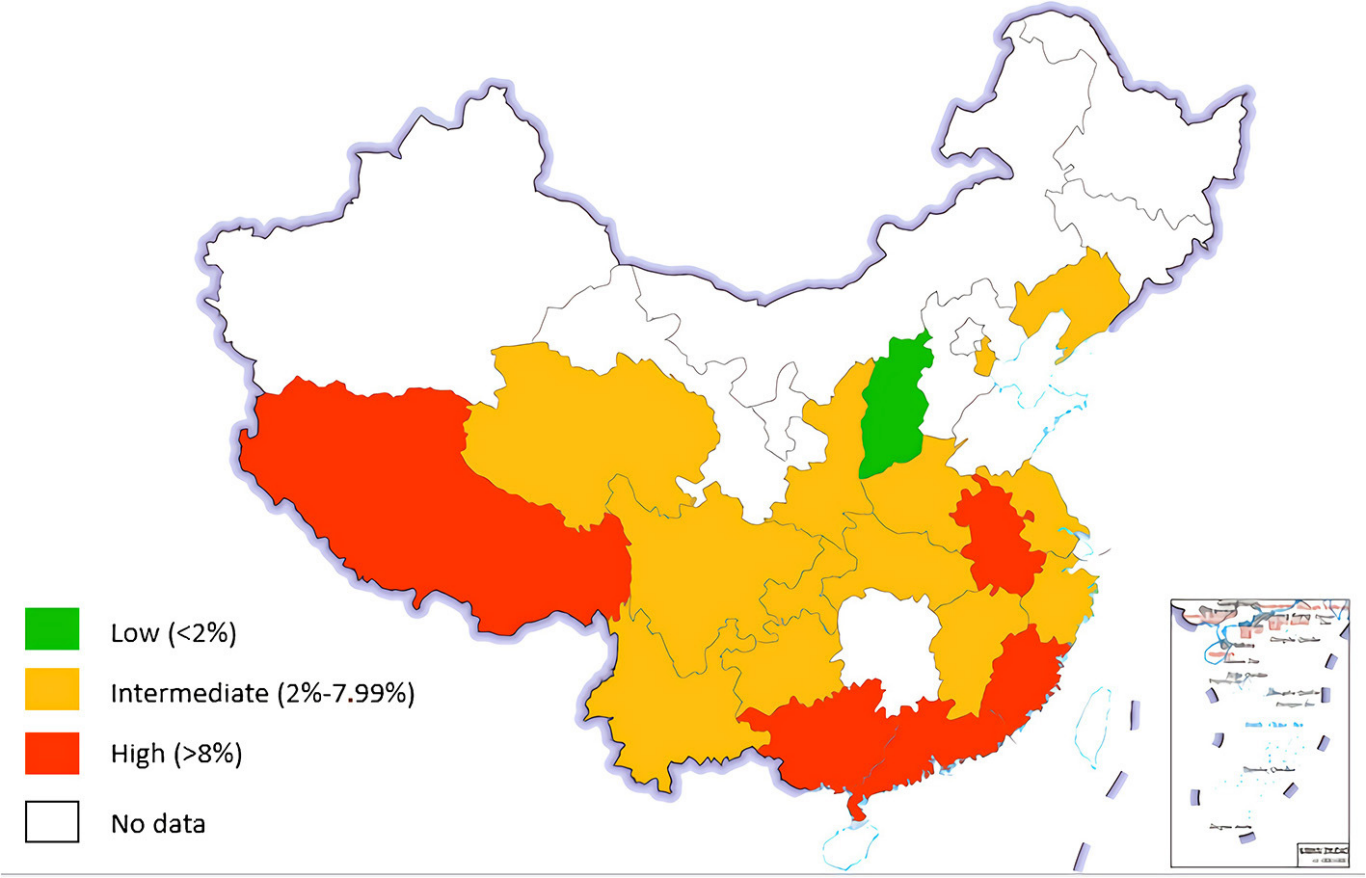
The prevalence of HBV infection among pregnant women by province in China, 2016–2021.

**Figure 4:**
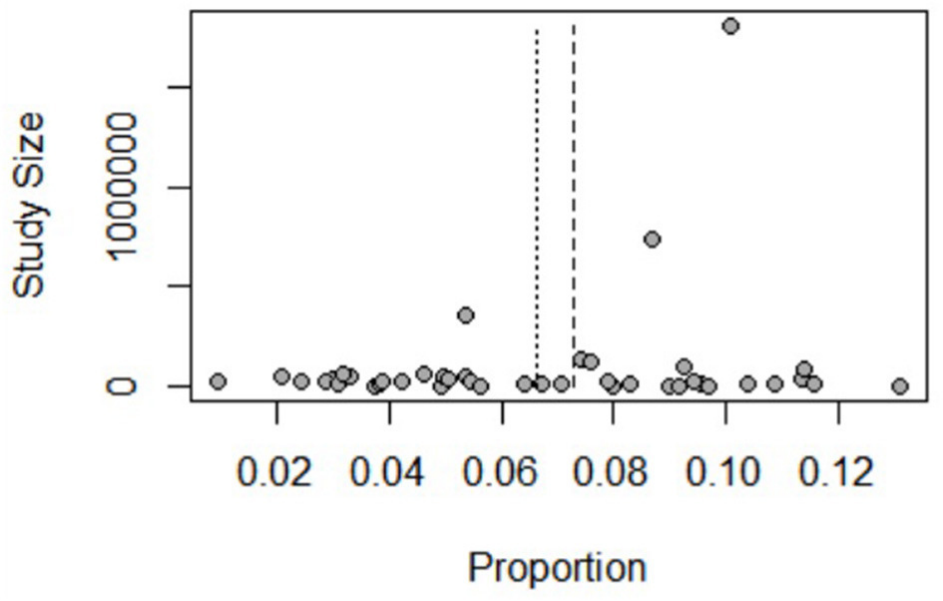
Bias assessment funnel plot of studies reporting HBV infection prevalence rate in Chinese pregnant women, 2016–2021.

The HBV prevalence in pregnant women in China was 6.64% (95% CI: 5.72–7.57%) during the period between 2016 and 2021 (Figure 2). The HBV prevalence varied from 0.95 to 13.10% across studies (Figure 3). Jinzhong City of Shanxi Province reported the lowest prevalence, and Ali City in Tibet reported the highest prevalence. The results revealed that one region was categorized as low endemic area (Shanxi Province), 14 regions were intermediate endemic areas (Jiangxi Province, Liaoning Province, Qinghai Province, Jiangsu Province, Tianjin City, Shanghai City, Henan Province, Yunnan Province, Zhejiang Province, Guizhou Province, Sichuan Province, Hubei Province, Chongqing City, and Shaanxi Province), and 5 regions were high endemic areas (Guangxi Province, Guangdong Province, Anhui Province, Tibet, and Fujian Province). When omitting any study or studies with a risk score ≤ 3 points, the sensitive analysis showed consistent results. The range of the pooled proportion from 6.51% (95% CI: 5.61–7.42%) to 6.78% (95% CI: 5.88–7.69%) was narrow. The funnel graph (Figure 4) and the results of Begg's test (*p* = 0.0131) and Egg's test (*p* = 0.0388) revealed potential publication bias.

Subgroup analyses were conducted based on geographical region, economic level, sample source, study design, and the results from risk of bias assessment (Table 3, Supplementary Figures S1–S4). None of these factors influenced HBV prevalence, which is consistent with the results of the meta-regression. However, the following trends were noticed across subgroups: (1) HBV prevalence during pregnancy was the highest in western China, followed by eastern China, and the lowest in central China; (2) regions with better economic levels were associated with higher HBV prevalence; (3) prevalence tended to be higher if the samples were collected from regions, compared with those from a single or multiple medical institutions.

**Table 3:** Subgroup analysis of included studies.

		**Numbers of Study**	**Proportion (95% CI)**	**I^**2**^**	***P***
Regional disparities	Western China	16	7.15% (95%CI 5.82–8.48%)	99.6%	0.4592
	Eastern China	20	6.63% (95% CI 5.20–8.06%)	99.9%	
	Central China	6	5.23% (95%CI 2.50–7.96%)	99.6%	
Economic level	1–10	24	6.78% (95% CI 5.66–7.90%)	99.9%	0.4968
	21–31	11	6.46% (95% CI 4.35–8.58%)	99.8%	
	11–20	7	6.64% (95% CI 3.87–8.58%)	99.9%	
Source of data	Regional investigation	11	7.48% (95% CI 5.69–9.27%)	99.9%	0.2906
	Medical institution	31	6.35% (95% CI 5.27–7.43%)	99.8%	
Study design	Cross-sectional study	35	6.65% (95% CI 5.71–7.59%)	99.9%	0.9606
	Cohort study	7	6.57% (95% CI 3.45–9.69%)	99.9%	

### Seroepidemiology Among HBsAg Positive Pregnant Women

Hepatitis B virus is more contagious when both HBsAg and HBeAg are positive, and when HBsAg is positive with high maternal concentrations of HBV DNA (>2 × 10^5^-1 × 10^6^ IU/ml). Twenty studies reported the testing results of HBeAg in pregnant women, and pooled data revealed that the HBeAg positive rate was 25.80% (95% CI: 22.26–29.69%, I^2^ = 98%) among HBsAg positive women. Moreover, the number varied from 12.14 to 43.71% across studies. The lowest HBeAg positive rate was found in Anqing City of Anhui Province, and the highest was in Guizhou Province. Subgroup analyses among different regions demonstrated that the HBeAg positive rate was 24.92% (95% CI: 19.76–30.92%, I^2^ = 98%), 20.79% (95% CI: 15.13–27.88%, I^2^ = 88%), and 30.07% (95% CI: 24.90–32.14%, I^2^ = 97%), respectively, in eastern, central, and western China.

Five studies reported the HBV DNA level in pregnant women. The pooled data indicated that among HBsAg positive pregnant women, the rate of high HBV DNA level was 26.73% (95% CI: 22.45–31.50%, I^2^ = 83%), and the number varied from 16.13 to 36.13% across studies. The rate of high HBV DNA level was the lowest in Shaanxi Province, and the highest in Qiandongnan Prefecture of Guizhou Province.

### HBV Prevalence and Demographic Information of Pregnant Women

Twenty-six studies reported the relationship between HBV prevalence and demographic information of pregnant women, which included age, residential address, education level, and occupation. However, whether these factors are related to HBV prevalence remains controversial. Due to the great variations in grouping methods and study designs across studies, a descriptive analysis was performed in this review.

Twenty-three studies focused on the association between age and HBsAg positive rate in pregnant women. Sixteen studies (sample size 1,138,852) suggested that HBsAg positive rate increased with age (12, 13, 17, 19, 20, 23, 24, 27, 28, 30, 31, 33, 36, 39, 40, 42). Two studies (sample size 3,947) revealed that HBsAg positive rate was not statistically different among various age groups (46, 53). Three studies (sample size 94,525) indicated that compared to those with HBsAg negative, HBsAg positive women were older (16, 49, 52). Two studies (sample size 132,254) demonstrated that the age was not statistically different between HBsAg positive and negative groups (32, 48).

Ten studies analyzed the relationship between the times of gestation and parity and HBsAg positive rate. Seven studies (sample size 954,199) suggested that HBV prevalence in primigravida and primiparas was lower (12, 13, 28, 32, 48, 49, 52), while the other three studies (sample size 45,248) showed no relevance (16, 33, 46).

Four studies presented the relationship between the education level and HBsAg positive rate. Two studies (sample size 782,262) showed that HBV positive rate decreased when education level improved (12, 32), and the other two studies (sample size 24,333) demonstrated no relevance (13, 16).

Five studies reported the HBV prevalence among pregnant women residing in rural or urban areas. Three studies (sample size 76,657) demonstrated that the HBsAg positive rate was higher in rural areas when compared to that in urban areas (27, 29, 46). However, two other studies (sample size 40,381) presented no statistical differences between rural and urban groups (33, 45). Moreover, two studies (sample size 755,378) showed that the HBsAg positive rate in local population was lower than that in migrant population (12, 20).

Two studies dug into the HBV prevalence among pregnant women with different occupations. All of them revealed differences. One of the studies (sample size 732,783) demonstrated the occupations with HBsAg positive rates from low to high as follows: staff members (8.27%), workers (8.52%), farmers (8.72%), home-based workers (9.17%), and businessmen (10.32%), successively (12). The other study (sample size 15,641) exhibited occupations with HBsAg positive rates from low to high as follows: company administrators (4.65%), farmers (5.41%), workers (7.87%), self-employed workers (7.94%), and unemployed population (9.38%), successively (28).

## Discussion

This study systematically searched literature published after 2016, which provided the baseline information of HBV infection among pregnant women in China. Liu et al. (7) revealed that the national HBV prevalence in Chinese pregnant women was 6.17% (95% CI: 6.16–6.18%). The number revealed in our study was 6.64% (95% CI: 5.72–7.57%), which was slightly higher than that from Liu's study. Similar differences were observed regarding regional HBV prevalence. Some possible reasons for the differences are as follows: (1) During 2016 and 2021, only 20 provinces had relevant studies reported, while other provinces such as Inner Mongolia, Beijing, Hebei, Shandong, Jilin, Xinjiang, Ningxia, Gansu, and Heilongjiang had no relevant studies conducted. Most of these provinces without relevant studies were intermediate endemic (2.00–4.99%). Missing of these data led to a high HBV prevalence in our study. (2) Since the establishment and implementation of health policies by the Chinese government in 1992, there has been significant progress in the decline of HBV prevalence (54). The prevalence data included in our study was published between 2016 and 2021, while the data in Liu's (7) study was collected in 2020, which is a later time period when compared with our study. The differences between the two study results were consistent with the decline in HBV prevalence. The same trend was observed in another systematic review published in 2013 (55), which showed a higher HBsAg positive rate (7.6%) among pregnant women when compared with our study.

Our study showed that the regions with HBV prevalence ranking from high to low were in western China, eastern China, and central China, successively, which was consistent with the findings from a systematic review of Chinese population in 2019 (56) and a national HBV serological study conducted in 2014 (57). However, Liu's study (7) showed that the highest prevalence was in eastern China. A possible reason for this difference was that almost no relevant studies were published in some western regions such as Inner Mongolia, Xinjiang, Gansu, and Ningxia. These provinces have low HBV prevalence. Missing of these data led to a high HBV prevalence in our study. The difference between regions might be caused by social factors. Relatively prosperous economy in the eastern region might bring more economic and social exchanges compared with other regions, which increased the risk of HBV transmission (58).

Our study showed that the positive rate of HBeAg (25.80%) is similar to the rate of high HBV DNA (≥2 × 10^5^-1 × 10^6^ IU/ml) (26.73%) in HBsAg positive pregnant women, which confirmed that the use of HBeAg test in place of HBV DNA test is reasonable. An observational study demonstrated that HBeAg positive rate among HBsAg positive couples was 27.84% in rural China, which decreased significantly with age (59). Both Liu's study and (59) our study demonstrated similar HBeAg positive rates among HBsAg positive pregnant women. Our study indicated that a great number of pregnant women with HBV infection carry high risks of transmitting infection, and special attention should be paid to block mother-to-child transmission in this population. Although the mother-to-child transmission rate declined from 50 to 6% in China since the implementation of HBV vaccination program, the rate remains high (11%) among pregnant women with positive HBeAg (4). In addition, pregnant women with positive HBsAg and HBeAg carry actively replicating virus, are more contagious, and prone to HBV mother-to-child transmission. Therefore, WHO recommends that pregnant women with a high HBV DNA (≥2 × 10^5^ IU/ml) should receive antiviral therapy during the last trimester of pregnancy in order to prevent mother-to-child transmission, and HBeAg test can be used as an alternative testing method in settings when antenatal HBV DNA test is not available (60).

The age, times of gestation and parity, residential address, and educational level of pregnant women may affect HBV prevalence, although no consensus was reached across studies. Further studies on these factors were demanded. HBsAg positive rate in pregnant women increased with age, which may be related to the weakening or subsiding of the protective effect from hepatitis B vaccine (12). It was suggested that women of childbearing age should be the prior recipients of hepatitis B vaccine. Additionally, the antibody level of the population should be monitored in real time and a booster dose should be given in time. The HBsAg positive rate of pregnant women living in urban areas or with higher education level is relatively low, which may be related to better access to health care and knowledge about hepatitis B transmission (21). It is important for public education on hepatitis B including its prevention and control methods, especially for populations with high risks for the disease.

Some limitations of our study were as follows. First, our study only included the HBV prevalence data of 20 provinces, and the studies were not distributed equally across regions. Due to the large differences in HBV prevalence among pregnant women in different regions, the situation in regions where no relevant studies were published remains unknown. Besides, there was potential publication bias as revealed by the funnel plot and Begg's and Egger's tests. However, these methods for detecting publication bias are based on the assumptions that when compared with studies with positive and/or significant results, small studies reporting negative results and/or small effects are less likely to be published (61). The studies included in our meta-analysis did not calculate significant levels for their results, accordingly, statistical non-significance is unlikely to be an issue that may have biased publications (62). Hence, the conclusion regarding the presence of publication bias should be drawn with caution. Lastly, due to the great variations in grouping methods and study designs across studies, meta-analysis for factors affecting HBV prevalence was not conducted, a descriptive analysis was performed instead.

## Conclusion

The prevalence of HBV in Chinese pregnant women is intermediate endemic (6.64%), although disparities exist between different regions. Among pregnant women with HBV infection, a high proportion of patients (25.80%) have strong infectivity, thus, effective mother-to-child blocking methods should be implemented, especially for populations with high risks for the disease. Moreover, factors affecting HBV prevalence among pregnant women remain controversial, and further research is needed to dig into the relationship between different factors and HBV prevalence.

## Data Availability Statement

The raw data supporting the conclusions of this article will be made available by the authors, without undue reservation.

## Author Contributions

DL, YL, and JN: study conduct. YL: data collection. HL and LinaZ: data analysis. JN, CZ, QY, and LiZ: data interpretation. DL and LingZ: drafting article. All authors contributed to the article and approved the submitted version.

## Funding

This work was supported by the National Natural Science Foundation of China [Grant No. 72074142].

## Conflict of Interest

The authors declare that the research was conducted in the absence of any commercial or financial relationships that could be construed as a potential conflict of interest.

## Publisher's Note

All claims expressed in this article are solely those of the authors and do not necessarily represent those of their affiliated organizations, or those of the publisher, the editors and the reviewers. Any product that may be evaluated in this article, or claim that may be made by its manufacturer, is not guaranteed or endorsed by the publisher.

## Abbreviations

CLIA: chemiluminescence immunoassay
CMIA: chemiluminescence micro particle immunoassay
ECLIA: electrochemiluminescence immunoassay
ELISA: Enzyme-linked immunosorbent assay
HBV: hepatitis B virus
HBsAg: hepatitis B surface antigen
HBeAg: Hepatitis B e Antigens
anti- HBs: Anti-hepatitis B surface antigen antibody
anti-HBc: antibody to hepatitis B core antigen
anti-HBe: Hepatitis B E antibody
HBV DNA: hepatitis B virus deoxyribonucleic acid
PRISMA: Preferred Reporting Items for Systematic Reviews and Meta-Analyses
TRFIA: time resolved fluoroimmunoassay
NR: not reported
WHO: World Health Organization.
