# Supplementary material for: Hepatitis B Infection Among Pregnant Women in China: A Systematic Review and Meta-Analysis
Source: Front Public Health. 2022 Apr 12;10:879289. doi: 10.3389/fpubh.2022.879289 (PMC9039058; doi:10.3389/fpubh.2022.879289)

Additional file: S1 Table Quality assessment of included cohort studies by the Newcastle-Ottawa Scale (NOS).

| **Study ID** | **Selection** | | | | **Comparability** | **Outcome** | | | **Overall scores** |
| --- | --- | --- | --- | --- | --- | --- | --- | --- | --- |
|  | **Representativeness of the exposed cohort** | **Selection of the non-exposed cohort** | **Ascertainment of exposure** | **Demonstration that outcome of interest was not present at start of study** | **Comparability of cohorts on the basis of the design or analysis** | **Assessment of outcome** | **Was follow-up long enough for outcomes to occur** | **Adequacy of follow up of cohorts** |  |
| Sun 2021^[32]^ | 1 | 1 | 1 | 0 | 1 | 1 | 1 | 1 | 7 |
| Zhao 2020^[49]^ | 1 | 1 | 1 | 0 | 1 | 1 | 1 | 1 | 7 |
| Tan 2016^[33]^ | 1 | 1 | 1 | 0 | 1 | 1 | 1 | 1 | 7 |
| Zhuang 2017^[52]^ | 1 | 1 | 1 | 1 | 1 | 1 | 1 | 1 | 8 |
| Cai 2019^[13]^ | 1 | 1 | 1 | 1 | 1 | 1 | 1 | 1 | 8 |
| Zhang 2020^[46]^ | 1 | 1 | 1 | 0 | 1 | 1 | 1 | 1 | 7 |
| Cui 2016^[16]^ | 1 | 1 | 1 | 1 | 1 | 1 | 1 | 0 | 7 |

Additional file: S2 Table Quality assessment of included cross-sectional studies by the Agency for Healthcare Research and Quality (AHRQ) Methodology Checklist.

| **Study ID** | **Define the source of information (survey, record review)** | **List inclusion and exclusion criteria for exposed and unexposed subjects (cases and controls) or refer to previous publications** | **Indicate time period used for identifying patients** | **Indicate whether or not subjects were consecutive if not population-based** | **Indicate if evaluators of subjective components of study were masked to other aspects of the status of the participants** | **Describe any assessments undertaken for quality assurance purposes (e.g., test/retest of primary outcome measurements)** | **Explain any patient exclusions from analysis** | **Describe how confounding was assessed and/or controlled** | **If applicable, explain how missing data were handled in the analysis** | **Summarize patient response rates and completeness of data collection** | **Clarify what follow-up, if any, was expected and the percentage of patients for which incomplete data or follow-up was obtained** |
| --- | --- | --- | --- | --- | --- | --- | --- | --- | --- | --- | --- |
| Bao 2017^[12]^ | Yes | Yes | Yes | Yes | No | Yes | No | Yes | No | No | Yes |
| Chen 2018^[15]^ | Yes | Yes | Yes | Yes | No | Yes | No | Yes | No | No | No |
| Gong 2017^[18]^ | Yes | Yes | Yes | Yes | No | Yes | No | No | No | No | No |
| Guo 2019^[19]^ | Yes | No | Yes | Yes | No | Yes | No | No | No | No | No |
| He 2019^[20]^ | Yes | Yes | Yes | Yes | No | Yes | No | No | No | No | No |
| Kang 2019^[24]^ | Yes | No | Yes | Yes | No | Yes | No | No | No | No | No |
| Liu 2020^[28]^ | Yes | Yes | Yes | Yes | No | Yes | Yes | Yes | Yes | Yes | No |
| Sheng 2018^[30]^ | Yes | Yes | Yes | Yes | No | Yes | No | No | No | Yes | Yes |
| Tang 2019^[35]^ | Yes | Yes | Yes | Yes | No | Yes | No | No | No | Yes | Yes |
| Wang 2020^[39]^ | Yes | No | Yes | Yes | No | Yes | No | No | No | No | No |
| Wang F 2019^[39]^ | Yes | No | Yes | Yes | No | No | No | No | No | No | No |
| Wen 2021^[41]^ | Yes | No | Yes | Yes | No | No | No | No | No | No | No |
| Wu 2016^[42]^ | Yes | No | Yes | Yes | No | Yes | No | No | No | Yes | No |
| Ying 2019^[43]^ | Yes | No | Yes | Yes | Yes | Yes | No | No | No | No | No |
| Zhang Y 2020^[48]^ | Yes | Yes | Yes | Yes | Yes | Yes | No | No | No | Yes | Yes |
| Zhong 2018^[50]^ | No | Yes | Yes | Yes | No | Yes | No | No | No | No | No |
| Zhou 2019^[51]^ | Yes | Yes | Yes | Yes | No | Yes | No | Yes | Yes | Yes | No |
| Zou 2020^[53]^ | Yes | No | Yes | Yes | No | Yes | No | No | No | No | No |
| Liu 2019^[27]^ | Yes | No | Yes | Yes | Unclear | No | No | No | No | Yes | No |
| Liang 2017^[26]^ | Yes | Yes | Yes | Yes | No | Yes | No | No | No | Yes | No |
| Wang A 2019^[37]^ | Yes | No | Yes | Yes | No | Yes | No | No | No | No | No |
| Li 2016^[25]^ | Yes | Yes | Yes | Yes | No | Yes | No | No | No | Yes | Yes |
| Sun 2017^[31]^ | Yes | No | Yes | Yes | No | Yes | No | No | No | No | No |
| Wei 2019^[40]^ | Yes | No | Yes | Yes | No | Yes | No | No | No | Yes | No |
| Wang 2018^[36]^ | Yes | No | Yes | Yes | No | Yes | No | No | No | No | No |
| Zhang 2016^[45]^ | Yes | Yes | Yes | Yes | No | Yes | Yes | No | No | Yes | No |
| Zeng 2019^[44]^ | Yes | No | Yes | Yes | No | Yes | No | No | No | Yes | No |
| Huang 2017^[22]^ | Yes | Yes | Yes | Yes | No | Yes | Yes | Unclear | No | Yes | No |
| Ma 2019^[29]^ | Yes | No | Yes | Yes | No | Yes | No | No | No | No | No |
| Huang 2020^[23]^ | Yes | No | Yes | Yes | No | Yes | No | No | No | Yes | No |
| Du 2021^[17]^ | Yes | No | Yes | Yes | No | Yes | No | No | No | No | No |
| Zhang Q 2020^[47]^ | Yes | No | Yes | Yes | No | Yes | No | No | No | No | No |
| Chen 2016^[14]^ | Yes | No | Yes | Yes | No | Yes | No | No | No | No | No |
| Hu 2018^[21]^ | Yes | No | No | Yes | No | No | No | No | No | No | No |
| Tang 2017^[34]^ | Yes | No | Yes | Yes | No | No | No | No | No | No | No |

Additional file: S figure1. Forest of subgroup by regional disparities


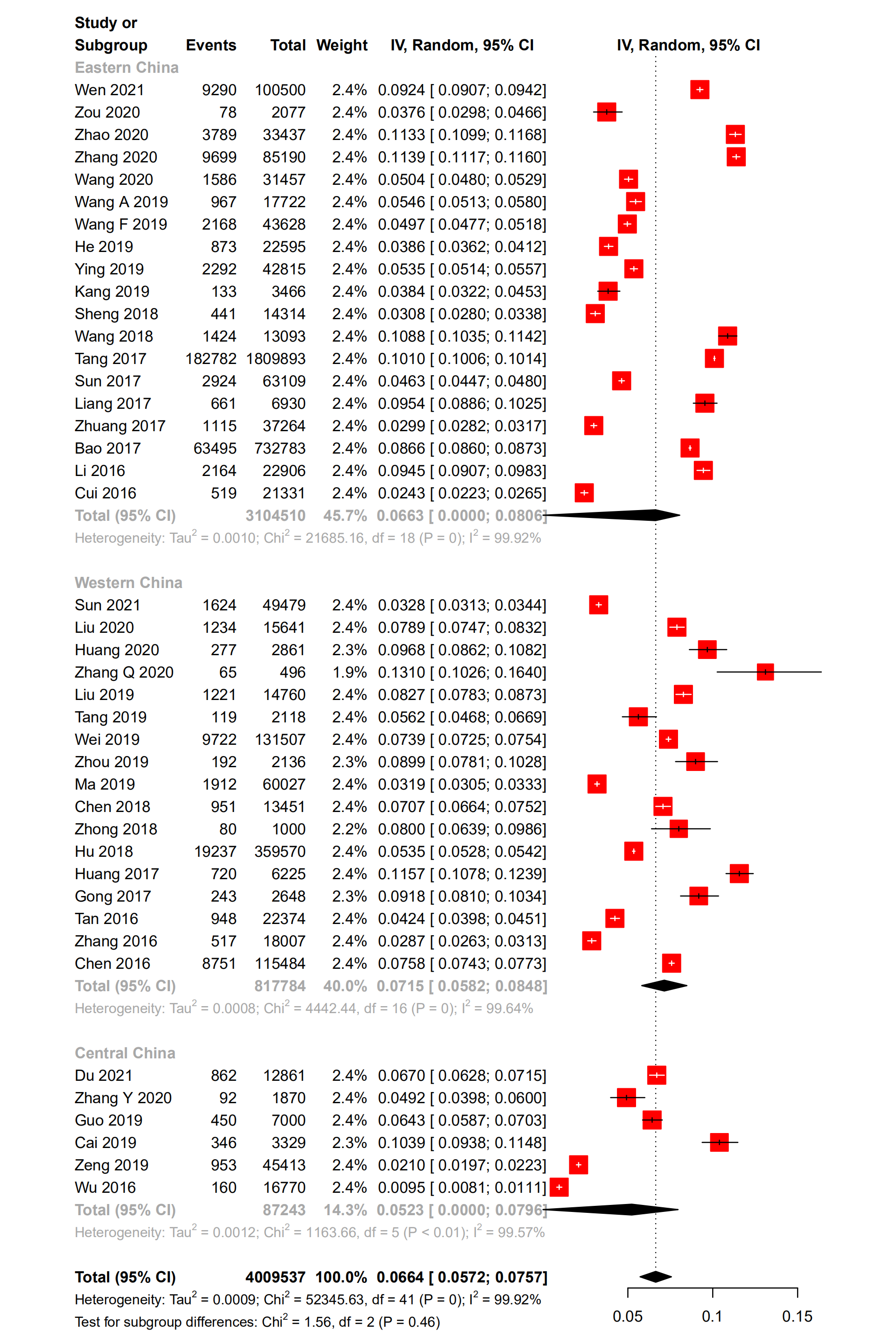


Additional file: S figure2. Forest of subgroup by [economic](javascript:;) [level](javascript:;)


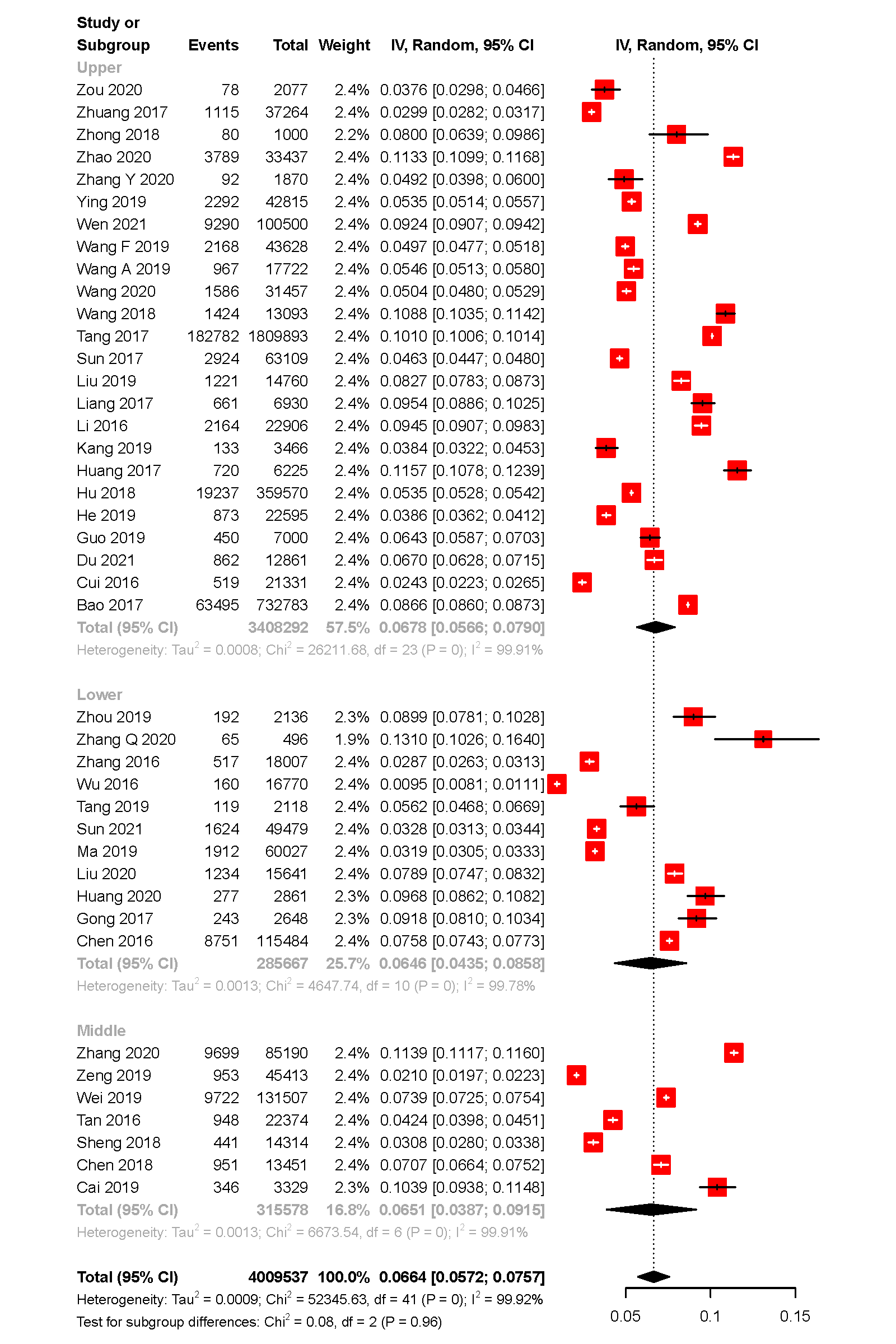


Additional file: S figure3. Forest of subgroup by source of data


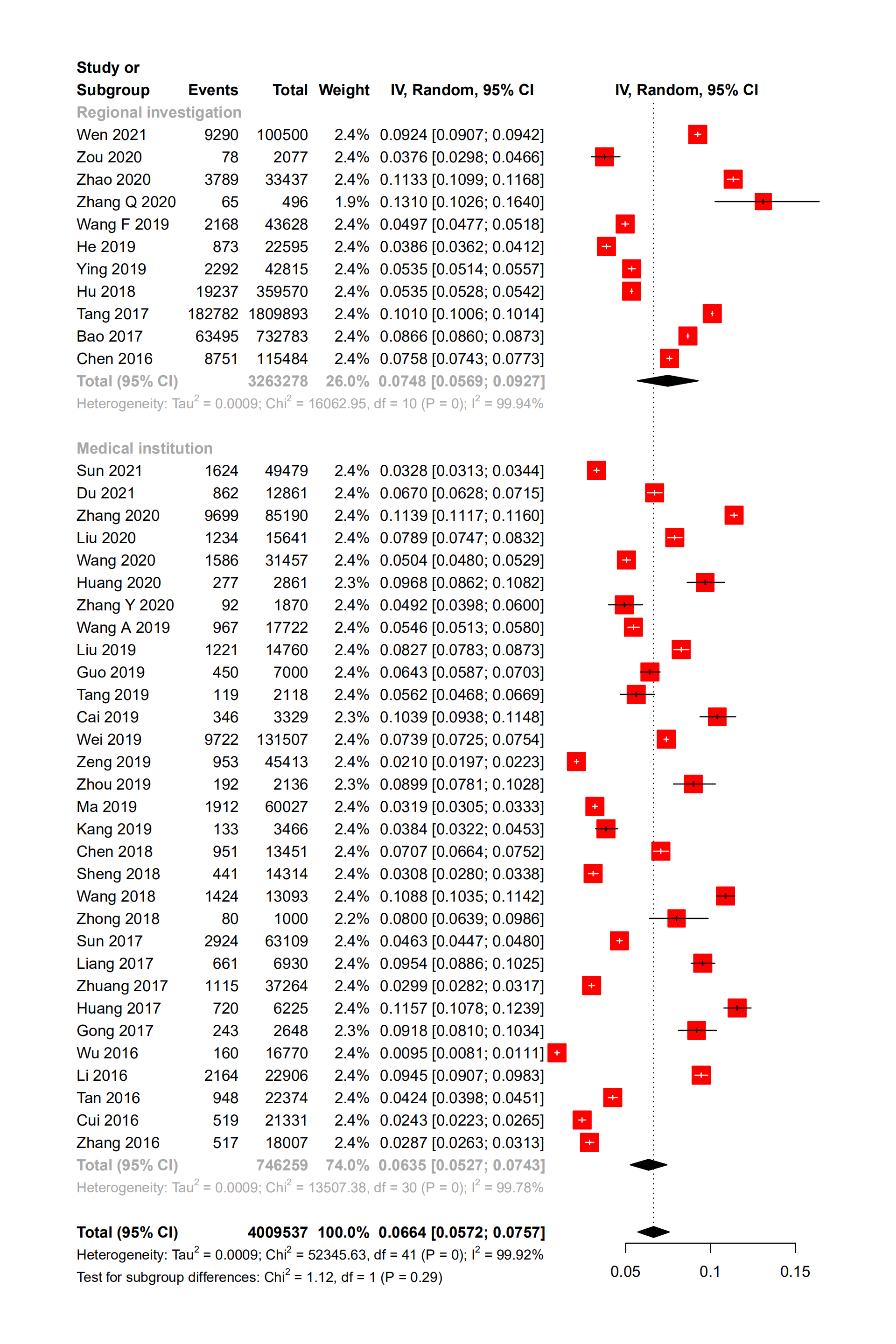


Additional file: S figure4. Forest of subgroup by study design


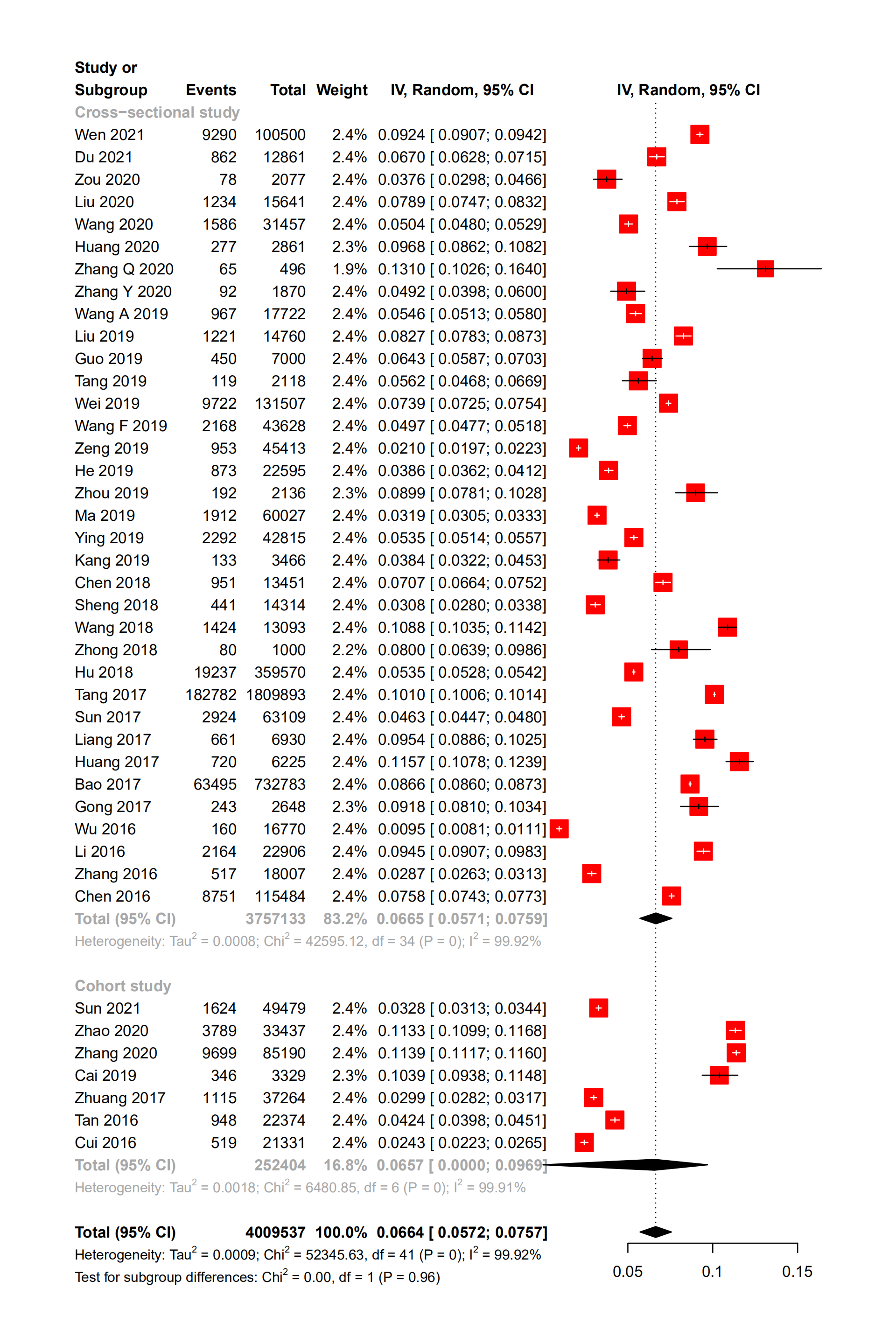

Supplement: Supplementary file 1 [file Data_Sheet_1.docx]
